# *Eremohadena afzalipouri* sp. nov. from Iran

**DOI:** 10.1673/031.012.13701

**Published:** 2012-11-26

**Authors:** Asghar Shirvani, Hadi Sheykhnejad, Mohammad Ali Shoghali

**Affiliations:** ^1^ Department of Plant Protection, Faculty of Agriculture, Shahid Bahonar University of Kerman, 76169-133 Kerman, Iran; ^2^ Young Researchers Society, Shahid Bahonar University of Kerman, Kerman, Iran; ^3^ No. 51, 24 Azar Street, 7619764755, Kerman, Iran

**Keywords:** checklist, Xyleninae

## Abstract

A new species of the genus *Eremohadena* Ronkay, Varga and Fabian, *Eremohadena afzalipouri* Shirvani sp. nov., is described from southeastern Iran. Holotype and female paratype and genitalia of both sexes are illustrated for the new species. A checklist of Iranian species of *Eremohadena* including nine species and subspecies is provided.

## Introduction

The genus *Eremohadena* Ronkay, Varga and Fabian, 1995 belongs to Xylenini, one of 12 tribes within the subfamily Xyleninae, as defined by Lafontaine and Fibiger (2006). Association of the genus with Xylenini is supported by one synapomorphy from male genitalia: the very large digitus, which forms a sclerotized area along the costal margin of the valva and is often partially or completely fused with the apical portion of the valva (Lafontaine and Fibiger 2006; Fibiger and Hacker 2007). Xylenini comprises five subtribes, of which Pseudohadenina includes six genera, containing *Pseudohadena* Alphéraky, 1889. The members of *Pseudohadena* genus-group were later revised and redefined by Ronkay et al. (1995), who suggested its division into three genera, including *Eremohadena* Ronkay, Varga and Fabian, and *Graphantha* Ronkay, Varga and Fabian, 1995.

The eremic genus, *Eremohadena*, currently comprises three subgenera, five species-groups, and includes 28 total species and subspecies (Fibiger and Hacker 2007). The members of this genus are univoltine (with flight period in late spring and autumn), and occur in lowlands, semi-mountains, and deserts (dry areas with poor vegetation) (Ronkay et al. 1995). Of the three subgenera, the newly discovered species belongs in *Eremohadena* Ronkay, Varga and Fabian, 1995. It is especially associated with the *chenopodiphaga* species-group, which currently includes seven species, *E. chenopodiphaga* (Rambur, 1832), *E. eibinevoi* Fibiger, Kravchenko, Li, Mooser and Müller, 2006, *E. halimi* (Milliére, 1877), and *E. immunda* (Ewersmann, 1842).

The purpose of this study is to describe a new species of the genus *Eremohadena*. External and genital characteristics of the new species are illustrated, described, and diagnosed compared with its closest congeners. A checklist of nine species and subspecies of *Eremohadena* from Iran is provided with their provincial distribution in the country.

## Materials and Methods

Adult specimens were collected using mobile light trap systems (powered by 12 volt batteries and 8 watt Black light UVB tubes) in Kerman province, Southeast Iran. Genitalia of both sexes were dissected, stained, and mounted following Fibiger (1997). Prepared genitalia slides were examined using binocular stereomicroscope (Olympus SZ60). Photographs of adult specimens and genitalia were taken by a Canon Power Shot A710 Digital Camera, using a stereomicroscope eyepiece for the genitalia. Terminology used for external and genital descriptions follows both Ronkay et al. (1995) and Fibiger and Hacker (2007). All specimens examined were deposited in the collection of Noctuidae, Department of Plant Protection, Shahid Bahonar University of Kerman, Iran.

## Results and Discussion

**Figure 1.**
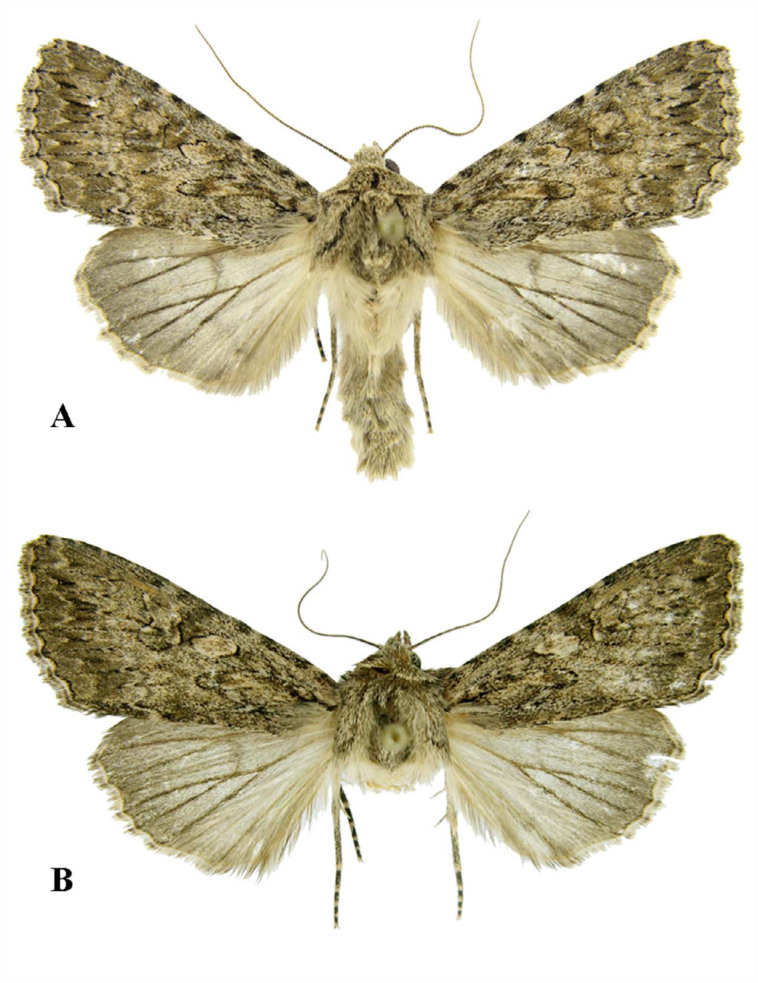
(A) *Eremohadena afzalipouri* sp. nov. Holotype, male. (B) *Eremohadena afzalipouri* sp. nov. paratype, female. High quality figures are available online.

**Figure 2.**
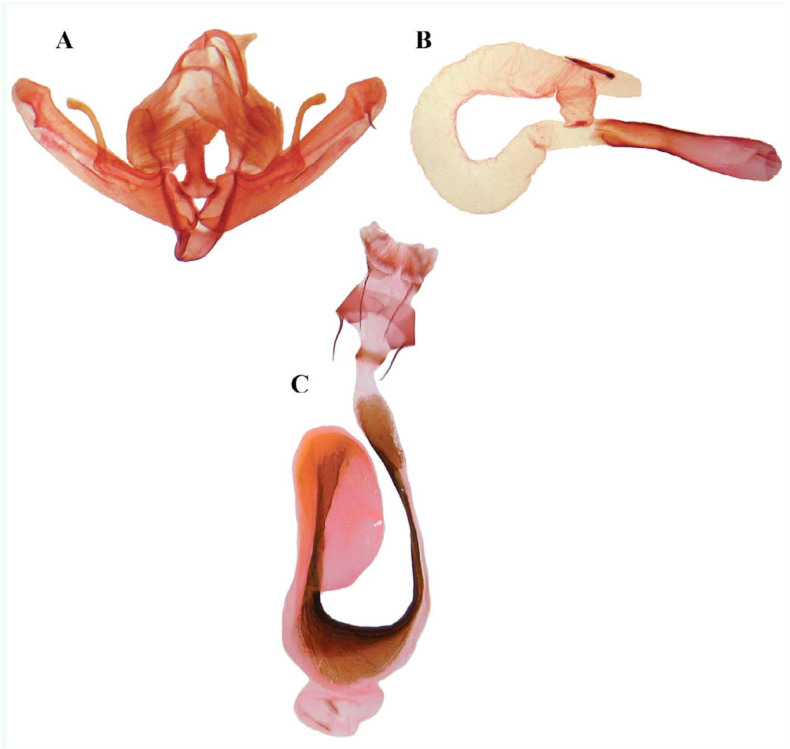
*Eremohadena afzalipouri* sp. Nov., genitalia. (A) Male armature; (B) Male aedeagus with everted vesica; (C) Female genitalia. High quality figures are available online.

**Taxonomy *Eremohadena afzalipouri* Shirvani sp. nov. (Figures 1, 2)**
**Description**
  Holotype: Male (Figure 1A). Wingspan 42–50 mm, thorax and abdomen strong, antennae ciliate, basally covered densely with white-cream scales, eye large, globular, palpi porrect, third segment short, one-third the length of the second segment; frons slightly bulged; head, collar, tegula and forewing ochreous light brown to brown-cream, pubescence of collar and tegula well defined, that of tegula outlined with black scales; tarsi ventrally armed with three rows of short and fine spines. Forewing large and triangular, dark brown costal spots present; small and narrow black basal dash present, crosslines well defined, antemedian line double, wedge shape, incomplete, postmedian line double, dentate; noctuid maculation complete, large, all encircled with fine black lines, orbicular stigma ovoid-irregular, lighter than ground color, reniform stigma ventrally filled with dark, darker path between two preceding stigmata, claviform stigma elongate; subterminal area more saturated in color, subterminal line defined as wedge-shape arrowheads, terminal line line, dark brown, fringes short, as ground color; underside of forewing cream, costal margin dirty light brown, veins well presented, discal spot present. Hindwing light brown-cream, marginal area dark, veins covered with brown scales, discal spot inconspicuous, fringes cream-beige, darker along veins; underside of hindwing dirty cream-white, discal spot present, postmedian line well defined and dark. Female (Figure 1B). Same size, wing coloration, and pattern as the male. Differs from the male by its filiform antennae.
  Male genitalia (Figure 2A, B). Uncus long, slender, basally curved then straight, hairy, apiculate; tegument narrow and high, penicular lobe elongate, large, reniform, densely hairy; juxta basally deltoid, medioapically long, parallel-side; valve long and narrow, broadest by sacculus, costal and ventral margins sclerotized, costal margin subapically with hump; digitus asymmetrical, that of right valve ending in an acute, narrow process, while that of the left side ending in a shorter thorn-like process; clavus missing, sacculus short, triangular, heavily sclerotized, editum wider than long, hairy; clasper parallel with valve margin, ampulla very long, apical one-third curved, spatulate; cucullus short, corona missing; vinculum v-shaped. Aedeagus long, basally dilated; vesica long and tubular, basally constricted, projecting laterally then coiled through a complete circle, subapically with finger-like diverticulum, apically with strong, spine-like cornutus.
  Female genitalia (Figure 2 C). Ovipositor moderate, cylindrical, almost weak, papilla anales apically with long hairs, rectangular, gonapophyses slender, posterior apophyses longer than anterior ones; ostium bursa short, sclerotized; ductus bursa slender, very long, distal end constricted and membranous, then slightly dilated and sclerotized, sclerotizsed bands lateral to the ductus bursa, which expand on the posterior end of the bursa sac and extend into the wall of the appendix bursa; appendix bursa elongate-ovoid; bursa sac globular, shorter than appendix bursa with two signum-stripes .
**Diagnosis**
  In comparison with *E. chenopodiphaga*, the ampulla of the new species is shorter and thicker, juxta and not triangular, and the vesica is thinner, with a complete coiling. Compared to *E. eibinevoi*, the vesica of *E. afzalipouri* sp. nov. is shorter and wider, the digitus of the right valve ending in a long, acute process. The characteristics of the female genitalia are even more diagnostic than the male's. The ductus bursa of *E. afzalipouri* sp. nov. is much longer and more slender, the ostium bursa is more sclerotized compared with *E. chenopodiphaga*, and the appendix bursa consists of only one sac.
**Type Materials**
  In total, six specimens were examined: Holotype: Male, Iran, Kerman province, Shahid Bahonar University, 1780 m.a.s.l., 30° 09′ 52″ N, 57° 09′ 06″ E, 02 May 2011, leg. H. Sheykhnejad, Slide No. AS545m. Paratypes: 1 male, 3 females, same location, 17 May 2010, 07 April 2010, 29 April 2010 (Slide No. AS476f), and 02 May 2011 (Slide No AS570f), leg. H. Sheykhnejad. One male, Iran, Kerman province, Joupar, 1870 m.a.s.l., 30° 03′ 44″ N, 57° 06′ 45″ E, 14 November 2009, leg. M. Shoghali, Slide No. AS480f.
**Biology and distribution**
  As other members of the genus *Eremohadena*, the new species also has an unusual life cycle, i.e., the presence of an aestivation phase. The moths fly in spring, then aestivate in summer, and then fly again in autumn. The specimens of *E. afzalipouri* sp. nov. were taken from two locations not far from each other, in the research farm of the College of Agriculture, Shahid Bahonar University of Kerman, in April and May 2011. The farm is located in a desert-lowland region surrounded by sandy lands with short-grass vegetation and tamarisk trees. One female specimen was collected in mid-November in the second location with similar vegetation. It seems that, like other congeners, this species is local, and not common. Adults are attracted to artificial lights. Immature stages and larval food plants, as for *E. eibinevoi*, are still unknown; however, the larvae of *E. chenopodiphaga* feed on foliage of *Chenopodium fructicosum*, *Atriplex portulacoides*, and *Salaola soda* plants (Fibiger et al. 2006).
  Etymology: The new species is named in honor of the late Alireza Afzalipour (1909–1993), founder of Shahid Bahonar University of Kerman.
  Checklist of Iranian *Eremohadena* and their local distribution in the country (* signifies the taxa have originally been described from Iran)
  Family Noctuidae
  Subfamily Xyleninae
  Tribe Xylenini
  Subtribe Pseudohadenina
  Genus *Eremohadena* Ronkay, Varga, and Fábián, 1995
  Subgenus *Eremohadena* Ronkay, Varga, and Fábián, 1995
  *coluteae* species-group
  *coluteae* (Bienert, 1869)*: Mazandaran (Ebert and Hacker 2002).
  syn. *arvicola* (Christoph, 1887)
  subsp. *banghaasi* (Bytinsky-Salz and Brandt, 1937)*: Tehran (Bytinsky-Salz and Brandt 1937).
  *siri* species-group
  *roseotinctoides* (Poole, 1989)*: Tehran, Mazandaran, Sistan va Balouchestan, and Kerman (Ebert and Hacker 2002).
  syn. *roseotincta* (Brandt, 1941) (preoccupied)
  *chenopodiphaga* species-group *chenopodiphaga* (Rambur, 1832): Tehran and Fars (Brandt 1938).
  *afzalipouri* Shirvani, sp. n.*
  *pexa* species-group
  *toerpexa* Ronkay and Gyulai, 2006*: Khorasan (Ronkay and Gyulai 2006).
  Subgenus *Iberihadena* Fibiger and Ronkay, 2007
  *immunis* (Staudinger, 1889): North of Iran (Hacker 1990).
  Subgenus *Megahadena* Ronkay, Varga and Gyulai, 2002
  *rjabovi* (Boursin, 1970)*: Tehran, Fars, Qom (Ebert and Hacker 2002), and Elburz Mountains (Boursin 1970).
  *megaptera* (Boursin, 1970)*: Elburz Mountains (Boursin 1970).
